# Effect of cerebrospinal fluid area mask correction on ^123^I-FP-CIT SPECT images in idiopathic normal pressure hydrocephalus

**DOI:** 10.1186/s12880-023-01038-x

**Published:** 2023-06-13

**Authors:** Makoto Ohba, Ryota Kobayashi, Chifumi Iseki, Kazukuni Kirii, Daichi Morioka, Koichi Otani, Yasuyuki Ohta, Yukihiko Sonoda, Koji Suzuki, Masafumi Kanoto

**Affiliations:** 1 Department of Radiology, Yamagata University Hospital, Yamagata, Japan; 2 Department of Psychiatry, Yamagata University School of Medicine, Yamagata, Japan; 3 Division of Neurology and Clinical Neuroscience, Department of Internal Medicine III, Yamagata University Faculty of Medicine, Yamagata, Japan; 4 Division of Diagnostic Radiology, Department of Radiology, Yamagata University Faculty of Medicine, Yamagata, Japan; 5 Department of Neurosurgery, Yamagata University School of Medicine, Yamagata, Japan

**Keywords:** ^123^I-FP-CIT, CSF area mask correction, DAT-SPECT, Idiopathic normal pressure hydrocephalus, Southampton method

## Abstract

**Background:**

Cerebrospinal fluid (CSF) area mask correction reduces the influence of low [^123^I]-N-fluoropropyl-2b-carbomethoxy-3b-(4-iodophenyl) nortropane (^123^I-FP-CIT) accumulation in the volume of interest (VOI) by CSF area dilatation on the specific binding ratio (SBR) calculated using the Southampton method. We assessed the effect of CSF area mask correction on the SBR for idiopathic normal pressure hydrocephalus (iNPH) characterized by CSF area dilatation.

**Methods:**

We enrolled 25 patients with iNPH who were assessed using ^123^I-FP-CIT single-photon emission computed tomography (SPECT) before shunt surgery or the tap test. The SBRs with and without CSF area mask correction were calculated, and changes in quantitative values were verified. Additionally, the number of voxels in the striatal and background (BG) VOI before and after CSF area mask correction were extracted. The number of voxels after correction was subtracted from that before correction, and the volume removed by the CSF area mask correction was calculated. The volumes removed from each VOI were compared to verify their effect on SBR.

**Results:**

The images of 20 and 5 patients with SBRs that were decreased and increased, respectively, by CSF area mask correction showed that the volumes removed from the BG region VOI were higher and lower, respectively than those in the striatal region.

**Conclusions:**

The SBR before and after CSF area mask correction was associated with the ratio of the volume removed from the striatal and BG VOIs, and the SBR was high or low according to the ratio. The results suggest that CSF area mask correction is effective in patients with iNPH.

**Trial registration:**

This study was registered in the UMIN Clinical Trials Registry (UMIN-CTR) as UMIN study ID: UMIN000044826. 11/07/2021.

## Background

[^123^I]-N-fluoropropyl-2b-carbomethoxy-3b-(4-iodophenyl) nortropane (^123^I-FP-CIT) single photon emission computed tomography (SPECT) visualizes the dopamine transporter (DAT) and is useful for diagnosing Parkinson’s disease (PD) and dementia with Lewy bodies (DLB) [1–3]. The findings of ^123^I-FP-CIT SPECT are mainly assessed visually [2, 4]; however, quantitative assessment is also used for ^123^I-FP-CIT SPECT image interpretation [5]. The specific binding ratio (SBR) for ^123^I-FP-CIT SPECT quantitation is calculated by subtracting the amount of ^123^I-FP-CIT accumulation in the striatal region from that of the whole brain. The accumulation in the striatal region is calculated as the ratio of the background (BG) volume of interest (VOI) [6]. The Southampton method described by Tossici-Bolt et al. [6] is widely used to calculate SBR.

However, when the region of cerebrospinal fluid (CSF) has expanded because of cerebral atrophy or ventricular enlargement, a CSF region of low ^123^I-FP-CIT accumulation can be mixed in the striatal VOI, which leads to an underestimation of the accumulated amount in the striatal VOI when applying the Southampton method [7]. Therefore, the CSF area mask correction method for masking the CSF area and calculating the SBR was developed [7]; this method has improved the ability to differentially diagnose patients with parkinsonism, such as PD and progressive supranuclear palsy (PSP) and those without parkinsonism, in cases with ventricular dilatation [8].

Idiopathic normal pressure hydrocephalus (iNPH) is characterized by cognitive dysfunction, gait disturbance, and urinary incontinence [9]. Furthermore, enlargement of the Sylvian fissures and ventricles and disproportional narrowing of the subarachnoid space and sulci in the higher fornix are hallmarks for diagnosing iNPH radiologically [9]. To date, the DAT imaging findings of iNPH have been reported to be normal because iNPH does not involve degeneration of the nigrostriatal neurons [10, 11]. However, it has recently been reported that DAT on DAT imaging was reduced in 30.8–62.0% of patients with iNPH [12–14]. Furthermore, different from PD showing DAT reduction in the putamen predominantly, iNPH may be characterized by DAT reduction in the caudate as much as the putamen [15]. In addition, PD and DLB often coexist with iNPH, and the coexistence influences DAT reduction [16]. Thus, DAT-SPECT imaging in iNPH might be clinically important for diagnosing and understanding its pathophysiology [15].

Idiopathic NPH presents with ventriculomegaly and is likely to include the CSF region in the VOI in the Southampton method. However, to the best of our knowledge, the effects of CSF area mask correction on SBR in iNPH have not been determined. We compared the SBR before and after CSF area mask correction in iNPH using ^123^I-FP-CIT SPECT and investigated the effect of the correction on the SBR.

## Methods

### Patients

The subjects were patients with iNPH who visited Yamagata University Hospital and were diagnosed based on the guideline diagnostic criteria [17] between March 2017 and April 2021. Of these, 25 patients with iNPH who were assessed using ^123^I-FP-CIT SPECT before shunt surgery or the tap test were included in this study. Furthermore, to establish the diagnosis of iNPH more reliably, only patients with an Evans index greater than 0.3 were included. This retrospective study was approved by the Ethical Review Committee of Yamagata University Faculty of Medicine (approval number: 2021-89).

The iNPH stage composition consisted of four possible cases, six probable cases, and 15 definite cases. All subjects in this study underwent ^99m^Tc-ethyl cysteinate dimer SPECT and ^123^I-metaiodobenzylguanidine (MIBG) myocardial scintigraphy, eliminating as much as possible DLB and PD, which are characterized by decreased blood flow in the occipital lobe [18] and/or decreased cardiac sympathetic function [18, 19]. The demographic and clinical data of the subjects are shown in Table 1.

**Table 1:** Demographic/clinical data and SBRs before and after CSF area mask correction

	Case	Age	Diagnosis	Evans index	Visual assessment	Removed Striatal VOI (pixels)	Removed BG VOI (pixels)	Differences in VOIs (Removed Striatal VOI-Removed BG VOI) (pixels)	ncSBR	cSBR	SBR change
Decreased SBR group	1	66	definite iNPH	0.46	Abnormal	1572	3059	-1487.0	5.03	4.87	0.15
	2	70	definite iNPH	0.32	Normal	1182	1947	-765.0	6.21	5.58	0.63
	3	71	definite iNPH	0.30	Normal	1712	2226	-514.0	5.12	4.87	0.26
	4	72	definite iNPH	0.35	Abnormal	946	2475	-1529.0	8.66	7.67	0.99
	5	73	definite iNPH	0.33	Normal	1025	2221	-1196.0	7.07	6.13	0.94
	6	77	definite iNPH	0.31	Normal	1314	2210	-896.0	6.58	6.08	0.50
	7	78	definite iNPH	0.31	Normal	1345	2122	-777.0	7.25	6.67	0.58
	8	81	definite iNPH	0.30	Normal	1372	1435	-63.0	5.77	5.51	0.26
	9	79	definite iNPH	0.31	Normal	1290	1813	-523.0	6.13	5.64	0.49
	10	79	definite iNPH	0.33	Normal	1733	1854	-121.0	6.52	6.48	0.04
	11	80	definite iNPH	0.30	Normal	1474	1739	-265.0	5.70	5.64	0.06
	12	81	definite iNPH	0.34	Normal	1636	2057	-421.0	5.51	4.96	0.54
	13	84	definite iNPH	0.35	Normal	1118	2376	-1258.0	6.07	5.27	0.81
	14	72	probable iNPH	0.35	Abnormal	1445	2384	-939.0	3.41	3.40	0.01
	15	75	probable iNPH	0.41	Normal	1931	2253	-322.0	8.46	7.78	0.68
	16	76	probable iNPH	0.31	Normal	1148	2645	-1497.0	6.68	5.66	1.02
	17	80	probable iNPH	0.35	Normal	1202	2042	-840.0	4.79	4.45	0.33
	18	83	probable iNPH	0.30	Abnormal	1410	2412	-1002.0	5.79	5.18	0.60
	19	78	possible iNPH	0.31	Normal	1304	2029	-725.0	6.04	5.80	0.24
	20	84	possible iNPH	0.30	Normal	1547	1852	-305.0	4.55	4.35	0.19
Increased SBR group	21	78	definite iNPH	0.38	Normal	2056	1763	293.0	4.45	4.87	-0.41
	22	83	definite iNPH	0.34	Normal	1933	1828	105.0	4.88	5.03	-0.14
	23	86	probable iNPH	0.36	Abnormal	1916	1426	490.0	2.41	2.88	-0.47
	24	80	possible iNPH	0.32	Abnormal	2197	1394	803.0	2.12	2.61	-0.49
	25	83	possible iNPH	0.41	Abnormal	2076	2140	-64.0	2.08	2.20	-0.12
Average (± SD)		78.0 ± 4.9	-	-	-	1515.4 ± 350.8	2068.1 ± 387.6	-	5.49 ± 1.71	5.18 ± 1.38	-

*SBR *Specific binding ratio, *CSF *Cerebrospinal fluid, *F *Female, *M *Male, *iNPH *idiopathic normal pressure hydrocephalus, *VOI *Volume of interest, *BG *Background, *ncSBR *the SBR without CSF area mask correction, *cSBR *the SBR with CSF area mask correction, *SD *Standard deviation, SBR change: the difference in SBR before and after CSF area mask correction, Removed Striatal VOI: volume of the striatal VOI removed by CSF area mask correction, Removed BG VOI: volume of the BG VOI removed by CSF area mask correction, Differences in VOIs: The volume difference in VOIs removed by CSF area mask correction

### SPECT imaging

We acquired ^123^I-FP-CIT SPECT/computed tomography (CT) images over 28 min using a Symbia T2 with a rotating, dual-detector gamma camera (Siemens Healthineers, Erlangen, Germany) and a low- to medium-energy general-purpose collimator (Siemens Healthineers), with 360° continuous rotation (7.0 min/rotation × 4 rotations). Supine patients at rest with their eyes closed were intravenously injected with ^123^I-FP-CIT (167 MBq), and SPECT/CT images were acquired for 3 h under the following conditions: magnification, 1.45; matrix, 128 × 128 (3.3 mm/pixel); main window, 159 ± 12.0 keV; and sub-window, 8%.

A Gaussian post-processing filter with a full width half maximum of 6.6 mm, six subsets, and eight iterations was applied for ordered subset expectation maximization reconstruction with CT-based attenuation correction, a multi-energy window, and collimator aperture correction for scatter correction.

### SBR calculation

**Fig. 1:**
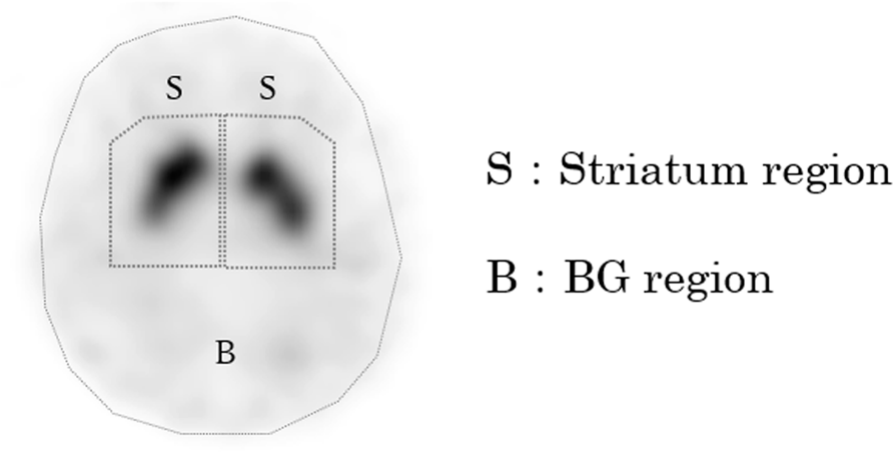
VOI for SBR calculation using the Southampton method. VOI (S): striatal region. VOI (B): BG region. VOI: volume of interest, SBR: specific binding ratio, BG: background

VOIs were placed on the striatum and BG regions determined using the Tossici-Bolt method [6] (Fig. 1). We obtained a highly reproducible quantitative index using a 44-mm-thick tomographic image centered on the striatum along with a large region of interest. SBR was calculated as follows:

$$\mathrm{SBR}={(\mathrm{Striatal}\;\mathrm{accumulation}-\mathrm{BG}\;\mathrm{accumulation})/\mathrm{BG}\;\mathrm{accumulation}}\times(\mathrm{Striatal}\;\mathrm{area}\;\mathrm{volume}/\mathrm{Striatum}\;\mathrm{actual})$$

### CSF area mask correction

The CSF area mask correction creates a standard normal distribution for each VOI in the striatal and BG regions and excludes the low accumulation of the set standard deviation (SD) from the SBR calculation. The SD threshold was set to an average of − 1.0 SD [7].

### Changes in SBR before and after CSF area mask correction

The SBR can be calculated using DaTView analysis (Nihon Medi-Physics, Tokyo, Japan), with differences between facilities corrected by applying the formula derived from the striatal phantom using SPECT/CT, y = 0.9369x + 0.0404 [20]. The SBRs with and without CSF area mask correction were calculated, respectively, and changes in the quantitative values were verified. The changes in SBRs were calculated as follows:

$$\mathrm{The}\;\mathrm{changes}\;\mathrm{in}\;\mathrm{SBR}=(\mathrm{SBR}\;\mathrm{without}\;\mathrm{CSF}\;\mathrm{area}\;\mathrm{mask}\;\mathrm{correction})-(\mathrm{SBR}\;\mathrm{with}\;\mathrm{CSF}\;\mathrm{area}\;\mathrm{mask}\;\mathrm{correction})$$

### Relationship between the changes in SBR before and after CSF area mask correction and the volume of the striatal VOI and BG VOI removed by CSF area mask correction

Removing a larger volume via CSF area mask correction results in a higher VOI count. Therefore, we hypothesized that the change in SBR was dependent on whether the volume removed by CSF area mask correction was larger in the striatal VOI or BG VOI. In other words, it was estimated that the SBR increased when the volume removed by the CSF area mask correction in the striatum VOI was larger than the volume removed by the CSF area mask correction in the BG VOI. Moreover, it was estimated that the SBR decreased when the volume removed by the CSF area mask correction in the striatum VOI was smaller than the volume removed by the CSF area mask correction in the BG VOI. The number of voxels in each VOI before and after CSF area mask correction was extracted from DaTView (Nihon Medi-Physics, Tokyo, Japan). The number of voxels after CSF area mask correction was subtracted from that before it, and the volume removed by the CSF area mask correction was calculated. The volumes difference between the striatal VOI and BG VOI removed by CSF area mask correction were calculated as follows:

$$\mathrm{The}\;\mathrm{volumes}\;\mathrm{difference}\;\mathrm{in}\;\mathrm{VOIs}\;\mathrm{removed}\;\mathrm{by}\;\mathrm{CSF}\;\mathrm{area}\;\mathrm{mask}\;\mathrm{correction}=(\mathrm{volume}\;\mathrm{of}\;\mathrm{striatal}\;\mathrm{VOI}\;\mathrm{removed}\;\mathrm{by}\;\mathrm{CSF}\;\mathrm{area}\;\mathrm{mask}\;\mathrm{correction})-(\mathrm{volume}\;\mathrm{of}\;\mathrm{BG}\;\mathrm{VOI}\;\mathrm{removed}\;\mathrm{by}\;\mathrm{CSF}\;\mathrm{area}\;\mathrm{mask}\;\mathrm{correction})$$

$$\mathrm{The}\;\mathrm{volumes}\;\mathrm{difference}\;\mathrm{in}\;\mathrm{VOIs}\;\mathrm{removed}\;\mathrm{by}\;\mathrm{CSF}\;\mathrm{area}\;\mathrm{mask}\;\mathrm{correction}\;\mathrm{were}\;\mathrm{compared}\;\mathrm{toverify}\;\mathrm{their}\;\mathrm{effect}\;\mathrm{on}\;\mathrm{the}\;\mathrm{SBR}$$

### Comparison of SBR before and after CSF area mask correction with a database of healthy controls

A database of Japanese healthy controls shows the upper and lower limits of the 95% prediction interval for each age [20]. We compared the SBR before and after CSF area mask correction with the normal SBR obtained from these controls.

### Statistical analyses

Spearman’s rank-order correlation was used to evaluate the relationship between the Evans index and the value of SBR change before and after CSF area mask correction. In addition, we analyzed the relationship between the changes in SBR before and after CSF area mask correction and the volume difference between the striatal VOI and BG VOI removed by CSF area mask correction by the same statistical analyses. Statistical analysis was performed using SPSS software (version 27 IBM, New York, USA), and a *P*-value of less than 0.05 was considered statistically significant.

## Results

### Changes in SBR before and after CSF area mask correction

**Fig. 2:**
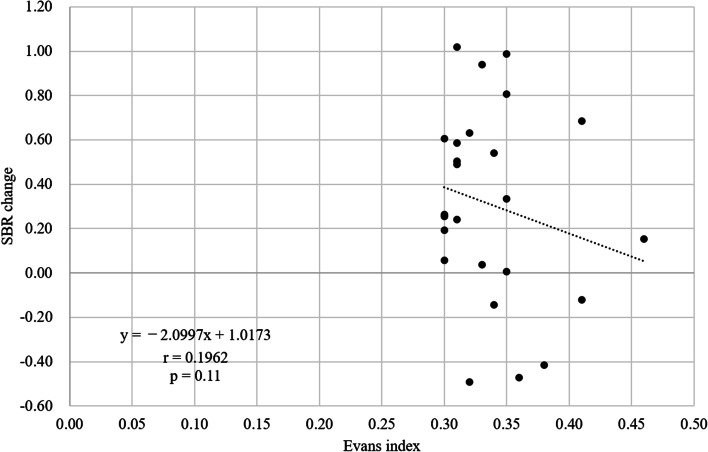
Relationship between the Evans index and the value of SBR change before and after CSF area mask correction. Scatterplot of the patients between Evans index and the value of SBR change before and after CSF area mask correction. There was no correlation between the Evans index and the value of SBR change before and after CSF area mask correction. The regression equations is y = –2.0997x + 1.0173 (r = 0.196, *p* =0.11). SBR: specific binding ratio, CSF: cerebrospinal fluid

The SBRs before and after the CSF area mask correction are shown in Table 1. The averages (± SD) were 5.49 ± 1.71 before correction and 5.18 ± 1.38 after correction. After CSF area mask correction, SBRs were low in 20 patients (cases 1–20) and high in five patients (cases 21–25). There was no correlation between the Evans index and the value of SBR change before and after CSF mask correction (r = − 0.196, *p* = 0.11) (Fig. 2.)

### Relationship between the changes in SBR before and after CSF area mask correction and the volume of the striatal VOI and BG VOI removed by CSF area mask correction

**Fig. 3:**
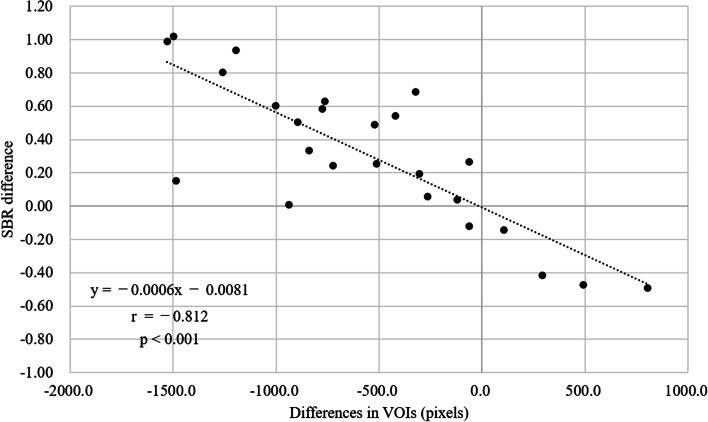
Relationship between the changes in SBR before and after CSF area mask correction and the volume of the striatal VOI and BG VOI removed by CSF area mask correction. Scatterplot of the patients between the voxel values obtained by subtracting the BG region from the striatal region removed by CSF area mask correction and the difference in SBR before and after the correction. A highly significant negative correlation was found between the difference in SBR before and after CSF area mask correction and the volume difference between the striatal VOI and BG VOI removed by CSF area mask correction. The regression equations is y = –0.0006x – 0.0081 (r = –0.812, *p* <0.001). VOI: volume of interest, SBR: specific binding ratio, BG: background, CSF: cerebrospinal fluid

The volumes of the striatal and BG region VOIs removed by CSF area mask correction are shown in Table 1. The images of 20 and five patients with SBRs that were decreased and increased, respectively, by CSF area mask correction showed that the volumes removed from the BG region VOI were higher and lower, respectively than those in the striatal region. Figure 3 shows the relationship between the difference in SBR before and after CSF area mask correction and the volume difference between the striatal VOI and BG VOI removed by CSF area mask correction. A highly significant negative correlation was found between those two values (r= − 0.812, *p* < 0.001).

### Comparison of SBR before and after CSF area mask correction with a database of healthy controls

**Fig. 4:**
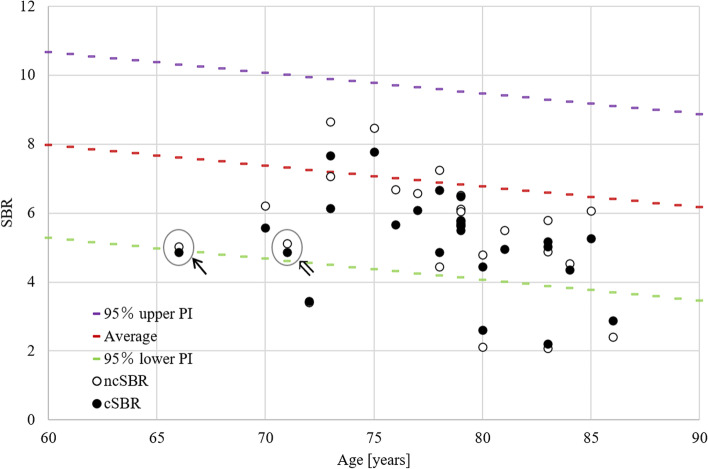
Comparison of SBR before and after CSF area mask correction with a database of healthy controls by age. Scatterplot of SBR before and after CSF area mask correction. Filled dots and white dots represent the SBR before and after CSF area mask correction, respectively. The dotted line shows the 95% upper (purple), average (red), and lower (green) prediction intervals of the Japanese healthy adult database. The cases in which SBR was below and near the lower limit of the 95% prediction interval after the CSF area mask correction were indicated by arrow (↖) and double arrow (⇖), respectively. SBR: specific binding ratio, CSF: cerebrospinal fluid, ncSBR: the SBR without CSF area mask correction, cSBR: the SBR with CSF area mask correction, PI: prediction interval

We compared the SBRs before and after CSF area mask correction using the phantom formula and the database of healthy controls by age (Fig. 4). The SBRs of one patient each were below and near the lower limit of the 95% prediction interval after the CSF area mask correction.

## Discussion

This is the first study to evaluate the SBRs before and after CSF area mask correction in patients with iNPH characterized by Sylvian fissures and ventricular enlargement, i.e., disproportionately enlarged subarachnoid-space hydrocephalus. Recently, a study assessing the effect of CSF mask correction in parkinsonian syndromes with ventriculomegaly, such as PD and PSP, has been reported [8]. In this prior study, CSF mask correction improved the ability to distinguish between patients with parkinsonism and those without parkinsonism in cases with moderate to severe ventricular dilatation [8]. This previous study has included a small number of iNPH patients in the group without parkinsonism. Recent studies have revealed that DAT availability decreases from 30.8 to 62.0% in patients with iNPH [12–14]. In other words, the results of discrimination in those studies may vary depending on the proportion of iNPH with and without the pathophysiology of DAT reduction if a large number of iNPH subjects were included.

Interestingly, changes in SBR with CSF mask correction in patients with iNPH were not constant in the present study. The SBR with CSF area mask correction was low in 20 patients and high in five patients compared with the uncorrected SBR. The changes in SBR were related to the ratios of the volumes removed by CSF mask correction from the striatal and BG regions. That is, individual differences in the enlargement of Sylvian fissures and the anterior horn of the lateral ventricle affected the SBR. The VOI analysis of the Southampton method is based on the premise that nonspecific accumulation in the BG regions is uniform. Therefore, CSF area mask correction might be particularly useful for diseases in which there are large individual differences in the enlargement of Sylvian fissures and cerebral ventricles.

It should be noted that the SBR after CSF area mask correction was below and near the lower limit of the 95% prediction interval of the normal database by age in one patient each. These findings suggest that CSF area mask correction might affect the visual and quantitative assessment of iNPH. The pathophysiology of DAT reduction, which was observed in approximately 30–60% of iNPH, has not been clarified [12–14]. Therefore, future studies of DAT availability in iNPH require more accurate visual and quantitative analyses. Consequently, we propose that CSF area mask correction should be applied when using the Southampton method in DAT imaging studies of iNPH.

This study had several limitations. First, we excluded patients with PD and DLB as much as possible based on cerebral blood flow SPECT/CT and MIBG myocardial scintigraphy; however, other iNPH-like disorders may have been included. Recently, a study reported that iNPH and α-synucleinopathy often coexist, with approximately 30% of iNPH cases meeting the diagnostic criteria for PD or PD with dementia and approximately 10% for DLB [16]. iNPH with α-synucleinopathy has lower SBR than iNPH without α-synucleinopathy [16]. Considering this coexistence of iNPH and α-synucleinopathy, the use of CSF area mask correction may become increasingly important. Second, complex pathologies, such as Alzheimer’s disease, which can affect cerebral atrophy, were not considered. Third, the study cohort was small, and more patients with definite iNPH were not available. Fourth, although the Evans index was used as an indicator of ventricular enlargement in this study, using other techniques to measure ventricular enlargement [21] may better validate the correlation between accurate ventricular volume and SBR. Fifth, we did not conduct a longitudinal study before and after shunt surgery. Recently, a case with iNPH has been reported in which abnormal DAT findings improved after shunt surgery, but quantitative analysis of SBR was not described [22]. Therefore, investigating the effect of CSF area mask correction on SBR when shunt surgery reduces ventricular enlargement may provide clinically useful information. Finally, this study was a retrospective study conducted at only one medical institution. Therefore, the results of this study should be validated in multicenter prospective studies.

## Conclusions

The SBR before and after CSF area mask correction was associated with the ratio of the volume removed from the striatal and BG VOIs, and the SBR was high or low according to the ratio. The results may indicate that CSF area mask correction is effective, particularly in patients with iNPH characterized by ventriculomegaly. In the future, further studies investigating the effect of CSF area mask correction for other neurodegenerative diseases are also needed.

## Data Availability

The data analyzed for this study can be provided by the corresponding authors on reasonable request.

## Abbreviations

^123^I-FP-CIT: [^123^I]-N-fluoropropyl-2b-carbomethoxy-3b-(4-iodophenyl) nortropane
BG: Background
CSF: Cerebrospinal fluid
DAT: Dopamine transporter
DLB: Dementia with Lewy bodies
iNPH: idiopathic normal pressure hydrocephalus
MIBG: Metaiodobenzylguanidine
PD: Parkinson’s disease
PSP: Progressive supranuclear palsy
SBR: Specific binding ratio
SPECT: Single photon emission computed tomography
VOI: Volume of interest
